# Rehabilitation of cognition and psychosocial well-being – a better life with epilepsy (ReCaP-ABLE): a protocol for a randomized waitlist-controlled trial

**DOI:** 10.3389/fneur.2023.1273550

**Published:** 2023-10-27

**Authors:** Kristijonas Puteikis, Asta Jakonienė, Arminas Jasionis, Peter Wolf, Rūta Mameniškienė

**Affiliations:** ^1^Faculty of Medicine, Vilnius University, Vilnius, Lithuania; ^2^Center for Neurology, Faculty of Medicine, Vilnius University, Vilnius, Lithuania; ^3^Danish Epilepsy Center Filadelfia, Dianalund, Denmark; ^4^Postgraduation Programme in Clinical Medicine, Federal University of Santa Catarina, Florianópolis, Santa Catarina, Brazil

**Keywords:** epilepsy, neuropsychology, memory, cognitive functions, quality of life, mental health, rehabilitation

## Abstract

Despite advances in the understanding of cognitive dysfunction among people with epilepsy (PWE), evidence for cognitive rehabilitation in epilepsy (CoRE) remains scarce. We present the protocol of a randomized waitlist-controlled trial (ClinicalTrials.gov ID NCT05934786) of a psychological-behavioral intervention aiming to ameliorate quality of life as well as cognitive functioning in a mixed PWE sample. The study is set at Vilnius University Hospital Santaros Klinikos and will offer adult PWE six individual and two group sessions led by a certified psychologist and directed toward improving memory, attention, self-regulation, mood and quality of life. The trial is expected to address major gaps in the literature by providing novel evidence on the effectiveness of CoRE in patients with genetic generalized epilepsies, the importance of epilepsy-specific factors for the response to CoRE, the impact of CoRE on long-term memory as well as its maintenance effects.

## 1. Introduction

Epilepsy is a multifaceted chronic neurologic disorder that occurs in every hundred individuals and directly affects patients’ cognition, quality of life as well as professional and societal activities (1). Because of premature mortality, mental health and socioeconomic implications of this disorder, it is now also recognized as a global public health priority by the World Health Organization (2). Cognitive dysfunction is a major burden for people with epilepsy (PWE) as epilepsy limits their ability to remember, learn, focus, and think. It has been shown that a third of newly diagnosed PWE have subjective cognitive complaints, and up to one half perform worse than controls during objective neuropsychological evaluation (3). Epilepsy can affect various cognitive domains, such as memory or attention, with a possible increase in the level of impairment over time (4). While the problem of prevalent cognitive dysfunction is well-known and may have significant impact on quality of life and social functioning in epilepsy, studies investigating the feasibility and efficacy of cognitive rehabilitation in epilepsy (CoRE) are rare: just nine group studies were identified in the most recent systematic review (5). Importantly, most were of only moderate quality. The lack of new studies of CoRE is seen as a significant shortcoming of modern epileptology: this neglect is thought to stem from a lack in resources and a historical focus on seizure control rather than cognitive outcomes in the clinical setting (6).

We aim to conduct a novel randomized waitlist-controlled cross-over trial of an original CoRE program, assess its overall efficacity and determine factors associated with a better response to this intervention. The main hypothesis of the trial is that a combined individual and group CoRE program is effective in improving quality of life and verbal as well as visual memory in PWE.

## 2. Methods and analysis

The study protocol is reported according to the “Standard Protocol Items: Recommendations for Interventional Trials (SPIRIT)” recommendations (7, 8). The SPIRIT checklist is provided as Supplementary Table S1.

### 2.1. Trial registration

The trial was registered at ClinicalTrials.gov as study No. NCT05934786 July 7, 2023. Items of the World Health Organization Trial Registration Data Set are presented in Table 1.

**Table 1:** Items of the World Health Organization Trial Registration Data Set.

Data category	Information
Primary registry and trial identifying number:	ClinicalTrials.gov ID NCT05934786
Date of registration in primary registry	2023-07-07
Secondary identifying numbers	P-MIP-23-333
Source(s) of monetary or material support	Research Council of Lithuania, agreement No P-MIP-23-333
Primary sponsor	Vilnius University (Principal Investigator – Rūta Mameniškienė)
Secondary sponsor(s)	Not applicable
Contact for public queries	Kristijonas Puteikis, kristijonas.puteikis@santa.lt
Contact for scientific queries	Rūta Mameniškienė, ruta.mameniskiene@santa.lt
Public title	Rehabilitation of Cognition and Psychosocial Well-being in Epilepsy
Scientific title	Rehabilitation of Cognition and Psychosocial Well-being – A Better Life with Epilepsy (ReCaP-ABLE): a randomized waitlist-controlled trial
Country of recruitment	Lithuania
Health condition studied	Epilepsy
Intervention	Behavioral: Cognitive rehabilitation Six individual one-hour therapy sessions with certified psychologists followed by two group sessions (a total of two months per patient). The intervention will consist of all parts of the Strategies-Outsourcing-Social support toolbox and include psychoeducation, lifestyle issues, coping strategies and homework.
Key inclusion and exclusion criteria	Inclusion criteria: Active epilepsy (medication for epilepsy and/or had at least one seizure in the past year). Adults (≥18 years). Lithuanian speakers. No intellectual disability. Exclusion criteria: Sensory or motor deficit preventing task completion. Epilepsy surgery planned during the project. Active non-paroxysmal comorbid disorder of the central nervous system (e.g., neurodegeneration, multiple sclerosis). Active psychiatric disorder during the past year. Psychoactive substance use (except social alcohol, tobacco and caffeine use).
Study type	Interventional randomized waitlist-controlled trial
Date of first enrollment	2024-01-01 (Estimated)
Sample size	70
Recruitment status	Not yet recruiting
Primary outcomes	Quality of life (Quality of Life in Epilepsy 31-item patient weighted version, QOLIE-31-P) 4 weeks post-intervention. Delayed verbal recall (Rey Auditory Verbal Learning Test, RAVLT) 4 weeks post-intervention. Delayed visual recall (Medical College of Georgia (MCG) Complex Figure test) 4 weeks post-intervention.
Key secondary outcomes	Symptoms of depression (Neurological Disorders Depression Inventory in Epilepsy, NDDI-E), anxiety (General Anxiety Disorder-7, GAD-7), stigma (Jacoby’s 3-item Stigma Scale).
Ethics review	Awaiting approval, Vilnius Regional Biomedical Research Ethics Committee
Completion date	2026-03-31 (estimated)
Summary results	Not applicable
Individual clinical trial participant-level data (IPD) sharing statement	No plan to share IPD

### 2.2. Study setting

The study will be set a tertiary epilepsy clinic of Vilnius University Hospital Santaros Klinikos (Vilnius, Lithuania) where patient recruitment and neuropsychological evaluation will take place. Patients will undergo CoRE at the Counseling and Training Center of the Faculty of Philosophy of Vilnius University.

### 2.3. Eligibility criteria

Patient enrolment and clinical evaluation will be done by a certified neurologist and include the following inclusion and exclusion criteria:

Inclusion criteria:

- Active epilepsy (medication for epilepsy and/or had at least one seizure in the past year).
- Adults (≥18 years).
- Lithuanian speakers.
- No intellectual disability.

Exclusion criteria:

- Sensory or motor deficit preventing task completion.
- Epilepsy surgery planned during the project.
- Active non-paroxysmal comorbid disorder of the central nervous system (e.g., neurodegeneration, multiple sclerosis).
- Active psychiatric disorder during the past year.
- Psychoactive substance use (except social alcohol, tobacco and caffeine use).

Patients with temporal lobe epilepsy as well as genetic generalized epilepsy will be enrolled.

### 2.4. Intervention

The intervention will consist of an eight-week-long psychological-behavioral program with six weekly individual sessions of 60 min followed by two group sessions. The group sessions are also planned to last 60 min and include five to seven PWE. The intervention will include all parts of the Strategies-Outsourcing-Social support toolbox (6) and involve psychoeducation, lifestyle issues, coping strategies, and homework (Box 1). The sessions will be led by certified psychologists, all of them will be trained by one leading specialist to ensure standardization. Patients will participate in group sessions led by the same specialist who provided individual sessions. There are no expected changes or modifications to the structure of the intervention upon its roll-out.

### 2.5. Outcomes

The primary outcome of the intervention will be measured by its effects (1) on quality of life and (2) memory function.

Changes in quality of life among PWE enrolled in the study will be estimated by comparing scores of the Quality of Life in Epilepsy 31-item inventory (patient weighted version, QOLIE-31-P) that has been validated in Lithuania and is among the most frequently used standardized quality of life assessment tools in PWE (9, 10). By selecting quality of life as the primary endpoint, we intend to detect broader effects of the intervention (i.e., beyond objective cognitive performance) representing direct benefits to participating PWE.

Verbal memory will be assessed by using the Lithuanian equivalent of the Rey Auditory Verbal Learning Test (RAVLT) that consists of five learning trials of a 15-word list A, one learning trial of a word list B and measuring the delayed recall of the word list after 30 min (11). Visual recall at 30 min will be measured by using the Medical College of Georgia (MCG) Complex Figure test for repeated testing (12).

**Figure 1:**
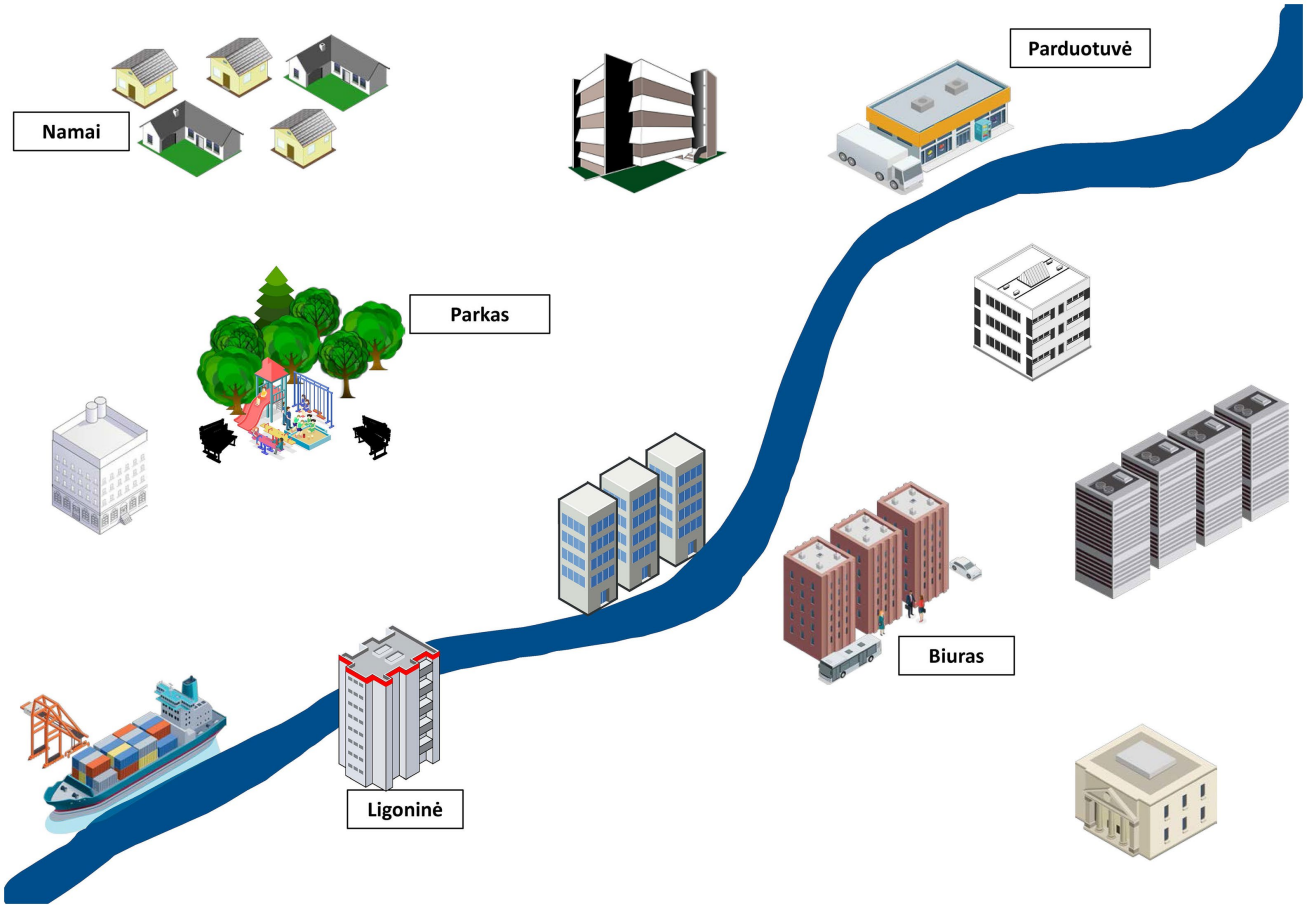
An example of a figure used in the experimental memory task. The figure will serve as a learning aid to memorize items of one week’s schedule, including place, time, and activity, as they are being read by the investigator. Participants will be asked to recall the same data after 30 min.

Secondary outcomes will include symptoms of depression (Neurological Disorders Depression Inventory in Epilepsy, NDDI-E) (13, 14), anxiety (General Anxiety Disorder-7, GAD-7) (15) and suicidality (Columbia Suicide Severity Rating Scale, C-SSRS) (16), metacognition (Metacognition Questionnaire-30, MCQ-30) (17, 18), Jacoby’s 3-item Stigma Scale (19), antiseizure drug adverse effects (Liverpool Adverse Events Profile, LAEP) (20, 21), health-related quality of life [the Short Form (36) Health Survey] (22) and subjective evaluation of cognitive functions (*ad hoc* Likert scales 0 to 10). Secondary cognitive outcomes will include reaction speed (Trail Making Tests A and B, Maze Task), working memory (Digit Span Test), verbal fluency (categorical and phonemic naming in 60 s), autobiographical memory (naming of recent personal autobiographical events), delayed verbal story recall as well as 1-week verbal recall to test for accelerated long-term forgetting. An experimental task set to test learning and recall of a hypothetical weekly schedule will also be used (Figure 1). This task was created by the authors and will be explored for applicability in testing for real-world event data, such as memory of where (e.g., in conference room 62B of the office), when (e.g., Monday at 15:30) and for what purpose (e.g., to be present in a business meeting) the participant is hypothetically planning to participate. The task will also include an item about preparatory actions before each activity (e.g., familiarize with material of the meeting in the latter example) and will be scored for each item recalled (maximum of 20 points). Given the exploratory nature of the task, it has not been previously validated and will rely on comparison between early and late intervention groups.

The possible learning effects at post-interventional follow-ups will be mitigated by using three different versions of the same memory tests as well as by comparing memory function in late vs. early intervention groups rather than improved performance in comparison to baseline scores in each subgroup.

BOX 1 **Outline of the content of the CoRE intervention.**

CoRE intervention plan (8 weeks)

1. Individual session.
  ■ Familiarization.
  ■ Goal setting. Discussion of the plan.
  ■ Presentation of the Mind-Emotion-Body connection.
  ■ Homework.
2. Individual session.
  ■ Discussion of homework.
  ■ Thought exercises.
  ■ Thinking training. Visual memory.
  ■ Homework.
3. Individual session.
  ■ Discussion of homework.
  ■ Body senses. Attention training.
  ■ Physiology training.
  ■ Homework.
4. Individual session.
  ■ Discussion of homework.
  ■ Working with emotions. Influence of emotions on quality of life and memory.
  ■ Emotion regulation.
  ■ Memory training.
  ■ Homework.
5. Individual session.
  ■ Discussion of homework.
  ■ Attention management.
  ■ Metacognition training.
  ■ Self-regulation.
  ■ Homework.
6. Individual session.
  ■ Discussion of homework.
  ■ Positive psychology.
  ■ Strengths and resources.
  ■ Memory training.
  ■ Homework.
7. Group session.
  ■ Discussion of homework.
  ■ The importance of social support.
  ■ Creating a circle of support.
  ■ Attention training.
  ■ Homework.
8. Group session.
  ■ Discussion of homework.
  ■ Compassion for self and others.
  ■ Memory and quality of life.
  ■ Homework. Summing up.
General plan of a 60-min session:
  ■ 10 min: presentation of the topic and discussion of homework.
  ■ 20 min: teaching of the topic.
  ■ 20 min: skill building.
  ■ 10 min: reflection. Questions. Homework.

Demographic (sex, age, educational and professional status, personal relationship status, socioeconomic status) and clinical (seizure type, epilepsy type, epilepsy etiology, epilepsy duration, seizure frequency, antiseizure medications used, seizure and electroencephalography laterality, localization of seizure focus (if present), handedness, somatic comorbidities) data of each participant will be collected to predefined case report forms.

### 2.6. Participant recruitment, allocation, and timeline

Study participants will be invited to participate in the trial during routine outpatient visits at the epilepsy clinic. They will have either temporal lobe or genetic generalized epilepsy as confirmed by the epileptologist, according to previously collected clinical (e.g., seizure semiology, patient history), instrumental (e.g., electroencephalography, video-electroencephalography), genetic and imaging (e.g., 3T magnetic resonance imaging) data required to substantiate the diagnosis according to guidelines by the International League Against Epilepsy. After acceptance, each new participant will be randomly assigned to either the early intervention group (EIG) or the late intervention group (LIG) at the time of enrolment by using open-source software for minimization (WinPepi) based on sex, epilepsy type and seizure control. The randomization will be done, and participants assigned to one of the groups by the principal investigator. Outcome assessors and data analysts will be blinded to participant status by using concealed patient coding and instructing patients not to discuss their status during examination. The principal investigator, psychologists performing the intervention and participants themselves will not be blinded.

**Figure 2:**
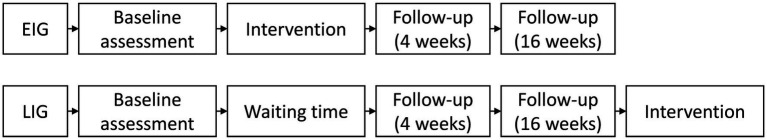
Timeline of the neuropsychological assessments and intervention. EIG, early intervention group; LIG, late intervention group.

Both the EIG and the LIG will undergo neuropsychological assessment at three time points (Figure 2). The EIG will be tested at baseline, with two follow-ups four and sixteen weeks after the intervention which itself lasts for eight weeks. The LIG will be tested at the same time points while on waitlist. Participants assigned to the LIG will be offered the intervention after the second follow-up. Both groups will receive otherwise routine clinical care (i.e., according to individual needs and best medical practice) at the tertiary epilepsy clinic. Given the non-invasive nature of the CoRE intervention, no adverse effects are expected. Therefore, discontinuation of the intervention is expected to occur only in the case of participant dropout. Patient attrition will be minimized by thoroughly discussing the aims and procedures of the trial before enrolment as well as by accommodating to the patients’ availability and daily schedule for the weekly sessions.

Encoded pseudonymized patient data will be collected by assessors in paper questionnaires and standardized assessment forms to be transferred to Microsoft Excel and saved in a National Open Access Scientific Data Archive Information System (“MIDAS”)^1^ designed to collect and keep different research data as well as to secure accessibility of data and information in a digital environment. Data quality will be preserved by checking all data fields for missing values or errors upon completion of assessment. This task will be done by the investigators of the trial without the need for an external data monitoring committee because of the relatively small study sample.

The study is expected to last from January 2024 (start of patient recruitment) to late March 2026 (end of the intervention for the LIG). No interim analyses are planned.

### 2.7. Sample size and statistical analysis

The target sample size of the study was calculated for a between-group interaction of a two-way repeated measures analysis of variance (ANOVA) with *f* = 0.40, *α* = 0.05, *β* = 0.95, two groups (early and late intervention), three measurement points, and 0.5 correlation between repeated measures. The resulting sample size of *n* = 58 (G*Power 3.1.9.7) was increased by 20% to *n* = 70 to account for dropouts (including the possibility of patient referral to presurgical evaluation if needed according to principles of best medical practice) and corresponded well to the mean sample size and attrition rates in previous trials (5). This sample size is deemed achievable as the trial will be conducted in a large university hospital covering tertiary epilepsy care services for approximately 1.4 million of inhabitants and include a group of PWE composed of patients with both TLE and GGE.

The efficacy of the intervention will be defined as statistically significant improvement on one of the primary outcomes (quality of life or delayed memory), tested with a repeated-measures between-factors analysis of variance (ANOVA) in the EIG as compared to the LIG. For secondary analyses, dynamic changes of other outcome measures will be tested, respectively, by using ANOVA or ANCOVA. The association between demographic and clinical variables with study endpoints will be conducted by means of linear and ordinal regression modeling. Subgroup analyses are planned to be conducted based on sex, education status, professional status, epilepsy type, laterality and lesionality. In case of missing data, multiple imputation will be used in sensitivity analysis.

## 3. Discussion

This protocol describes a planned randomized waitlist-control trial of CoRE in epilepsy. The study was designed to address major research gaps identified through recent systematic literature reviews in this field, as discussed below (5, 6, 23).

First, the study will include patients with genetic generalized epilepsy (GGE). Most trials examined CoRE by including patients with temporal lobe epilepsy (TLE), often in the context of presurgical evaluation (5, 24–26). As epilepsy surgery is not indicated in GGE, these patients are less frequently tested for neuropsychological deficits and have long been thought to have nearly normal cognitive functions. However, recent studies show frequent cognitive dysfunction in GGE as well (27). Their inclusion is expected to help define the efficacy of CoRE when there is no indication that seizure onset is focal (as is expected in both lesional or non-lesional TLE). While the inclusion of patients with GGE makes the study sample more heterogeneous than if only patients with TLE were enrolled, we believe there will remain opportunities to detect the effects of CoRE on different types of epilepsy through subgroup analysis in case of a large effect size.

Second, we are planning to investigate epilepsy-associated factors in response to cognitive rehabilitation beyond seizure laterality. Only two of the previous trials examined the impact of background patient epilepsy-related variables (e.g., seizure frequency, polytherapy, epilepsy onset time) on the efficacy of CoRE (5, 26, 28). This information will be gathered through standardized patient forms and included in secondary analyses.

Our project also includes novel measures of cognitive assessment. Baseline and follow-up assessments will consist of both traditional and experimental neuropsychological tools. While traditional instruments will ensure the comparability of the results with previous studies, novel tools will be essential to address the need to train and test ecologically valid everyday cognitive functions. We selected to test memory of a week’s schedule of daily activities – the task is expected to depend on attention, short-term visual and verbal memory as well as associative learning and transfer effects. Furthermore, we envision evaluating patients for accelerated long-term forgetting – to the best of our knowledge, the effects of CoRE on long-term memory deficits have not been investigated in earlier studies (29).

Moreover, the assessment we suggest includes testing for mental health status, metacognition and quality of life in addition to objective cognitive performance. The psychosocial status of the patient will be evaluated to adjust for subclinical levels of anxiety and depression as well as to see whether CoRE may improve patient mental health. Moreover, we will also investigate suicidality – this part of the evaluation is rarely done in the clinical setting and is especially important in Lithuania, which has extremely high suicide rates (14). Patients will also complete a metacognition questionnaire – a relatively novel tool in PWE set to assess coping and thinking mechanisms that underlie self-regulation in psychopathology and may help to explain better response to CoRE (17, 18). Finally, patient-oriented outcome measures (i.e., quality of life) will be essential to define the overall impact of the CoRE program (30, 31).

To increase the likelihood of the efficacy of the tested intervention, it will be done by following the S.O.S. toolbox: Strategies (internal and external), Outsourcing (use of physical and digital media) and Social support (education and co-operation) (6, 32). The intervention includes elements of psychoeducation mindfulness, positive psychology and acquires intensity from weekly homework that makes the program a continuous process that is not limited to the sessions themselves. The intervention will combine compensatory and restitution techniques alongside focus on general mental well-being and self-regulation. Because of such a varied inventory within the CoRE program, we expect it to have transfer effects for domains that will not be trained directly (e.g., long-term memory) (28).

Finally, our trial includes a longer follow-up period: while most previous studies had a limited follow-up period of 12 weeks, our timeline will include a follow-up of 16 weeks and provide a better estimate of the maintenance effects of CoRE (5).

## 4. Limitations

Despite the advantages of the planned study mentioned above, some of its limitations should be considered as well. First, the study will be of a single-country and single-center design, imposing boundaries on the sample size, generalizability of the study findings as well as the application of the intervention in different socioeconomic and cultural backgrounds. Second, the PWE group is expected to be heterogeneous in epilepsy type. This limitation will be addressed through subgroup and adjusted analyses. Third, the intervention will be focused on improving cognitive strategies rather than directly training selected cognitive domains. This may decrease the perceived effectiveness of the CoRE program on objective cognitive functioning as the effects of near transfer in such rehabilitation remain unknown. Finally, despite a longer follow-up than in other studies, the understanding of any emerging maintenance effects will remain limited to a relatively short period of 4 months.

## 5. Dissemination

Open access publishing of the study results will be given priority. The raw anonymized dataset is planned to be made available after publishing the results of the study upon reasonable request by third parties. The key to decode pseudonymized data will be available only to the principal investigator in physical format. Study results will also be disseminated through meetings with policy makers as well as in plain language articles in patient community websites and public press.

## 6. Conclusion

In this protocol we outlined a plan to conduct a randomized waitlist-controlled trial exploring the effects of a psychological-behavioral cognitive rehabilitation program on the quality of life and memory function in adults with epilepsy. This trial is an attempt to demonstrate feasibility and test the effectiveness of CoRE in a mixed PWE sample as well as to provide additional evidence about the target population for CoRE and the determinants of its effects. We believe that such an initiative will help further translate the experience that has emerged from neuropsychological evaluation in epilepsy to non-invasive add-on rehabilitation programs addressing burdensome cognitive issues among PWE.

## Ethics statement

Ethical review and approval for the study is sought from the Vilnius Regional Bioethics Committee that is overseeing all biomedical studies conducted in the Vilnius region. The Committee is responsible for the independent supervision of research conduct. The trial may also be subject to internal auditing by the administration of Vilnius University Hospital Santaros Klinikos. The study will be conducted with respect to the principles of the World Medical Association Declaration of Helsinki and each participant will provide written informed consent upon enrolment.

## Author contributions

KP: Conceptualization, Methodology, Visualization, Writing – original draft, Data curation, Formal analysis, Validation. AsJ: Methodology, Writing – review & editing. ArJ: Conceptualization, Methodology, Writing – review & editing. PW: Supervision, Writing – review & editing. RM: Conceptualization, Methodology, Visualization, Writing – original draft, Funding acquisition, Project administration, Supervision, Writing – review & editing.

## Footnotes

^1^ https://www.midas.lt/public-app.html#/apie/about?lang=en
